# Distributed multi-robot active gathering for non-uniform agriculture and forestry information

**DOI:** 10.3389/fpls.2025.1699124

**Published:** 2025-10-22

**Authors:** Jun Chen, Mingjia Chen, Jun Wang, Qi Mao, Fei Xie, Philip Dames

**Affiliations:** 1 Wenzhou Vocational College of Science and Technology, Wenzhou, China; 2 Wenzhou Key Laboratory of AI Agents for Agriculture, Wenzhou, China; 3 School of Electrical and Automation Engineering, Nanjing Normal University, Nanjing, China; 4 Department of Mechanical Engineering, Temple University, Philadelphia, PA, United States

**Keywords:** multi-robot systems, active information gathering, Thompson sampling, multi-target tracking, distributed control

## Abstract

Active information gathering is a fundamental task in multi-robot systems in agriculture, with applications in precision planting and sowing, field management and inspection, intelligent weeding and pest control, etc. Traditional distributed strategies often struggle to adapt to environments where information of interest are unevenly clustered, leading to slow detection and inefficient coverage. In this paper, we reformulate the information gathering problem as a multi-armed bandit (MAB) problem and propose a novel distributed Bernoulli Thompson Sampling algorithm. Our approach enables robots to make exploration-exploitation decisions while sharing probabilistic information across the team, thus improving global coordination without centralized control. We further combine the distributed Bernoulli Thompson Sampling policy with Lloyd’s algorithm for dynamic target tracking and introduce a goal swapping strategy to improve task allocation efficiency. Extensive simulations demonstrate that our method significantly outperforms baseline approaches in terms of search speed and target coverage, particularly in scenarios with clustered target distributions.

## 1 Introduction

Information gathering plays an essential role in agriculture, including fruit picking and farmland monitoring (Ma et al., 2025). Multi-robot information gathering enhances the quality of agricultural products while reducing labor costs by advancing agricultural automation and precision farming technologies (Mao et al., 2021). Effective operation in such settings relies on two fundamental components: an estimation module that detects and tracks the dynamic state of multiple targets, and a motion control module that coordinates robot motion for efficient target exploration and tracking. However, in environments characterized by sparse observations or spatially clustered targets, conventional estimation-control frameworks often lead to inefficient exploration and delayed target acquisition, limiting overall system performance.

### 1.1 Multi-target state estimation

We focus on the set of problems where robots must detect and track a large number of discrete objects (e.g., people, vehicles, landmarks), which is often modeled as a multi-target tracking (MTT) problem. Different from single target tracking, the main challenge of MTT is matching detections to target tracks, especially in the precense of false negative and false positive detections, a process known as data association. There are a number of standard MTT algorithms, each of which solve data association in a different way: global nearest neighbor [GNN; (Konstantinova et al., 2003)], joint probabilistic data association [JPDA; (Hamid Rezatofighi et al., 2015)], multiple hypothesis tracking [MHT; (Blackman, 2004)], and particle filters (Doucet et al., 2002). Each of these trackers propagates the posterior of target states over time and solves the data association problem prior to tracking. Learning-based methods such as the graph neural network [GNN; (Yang et al., 2022)] have also been shown to solve the data association problem in dense scenes.

Another class of MTT techniques, derived from random finite set (RFS) statistics [RFS; (Mahler, 2007)] simultaneously solves both data association and tracking. We use the probability hypothesis density (PHD) filter (Mahler, 2003), which tracks the spatial density of targets. This approach is best suited for situations where targets do not require a unique identity., *e.g.*, a rescue robot only needs to know where all of the people are located. Our previousv work developed a distributed PHD filter that is provably equivalent to the centralized solution (Dames, 2020).

### 1.2 Sensor-based control of MRSs

While simultaneous search and tracking for information that remains static over time is well-studied (Papaioannou et al., 2019), unknown and time-varying number of moving targets still leave challenge for MRSs. Lloyd’s algorithm (Lloyd, 1982) is one of the best-known control algorithms for distributed target search and tracking, the idea of which is to represent target states by a weighting function over the task space and to drive each robot to the weighted centroid of its Voronoi cell (Cortes et al., 2004). In our prior work, we use the PHD as the weighting function to realize sensor-based control of MRSs, driving robots to actively track targets (Dames, 2020). However, when no target is within a robot’s Voronoi cell, the robots move erratically, reacting to any false positive detections as well as the dynamically changing shape of their Voronoi cells. As a result, robots often stay within empty sub-regions instead of purposefully seeking out untracked targets, slowing down the rate at which they find targets. This problem is further exacerbated when a majority of targets gather within some small subsets of the environment, as is often the case in real life, *e.g.*, animals cluster around water sources within large nature reserves.

Our recent work (Chen et al., 2025b) develops a cumulative state estimation strategy to build-up a long-term belief of target distribution alongside the instantaneous state estimation, i.e., the PHD. By weighting each cell with the short- and long-term belief, robots tend to gather around areas with dense information. Yet, in this paper, we further investigate a lighter and more straight forward algorithm that can trade-off between exploring sparse areas and exploiting dense areas without relying on maintaining full cumulative posterior map over the entire task space.

One way to trade-off is to have idle robots (*i.e.*, those not tracking targets) sample the task space in a way that balances between searching low-density areas for undetected targets (exploration) and high-density areas to increase the probability of finding a target (exploitation). This coincides with the multi-armed bandit (MAB) problem (Auer et al., 2002), in which a gambler must decide which arm of. nonidentical slot machines to play to maximize the reward. MABs have been applied to multi-robot task allocation (Pini et al., 2012) and sensing (Luo and Sycara, 2018; Best et al., 2019; Shi et al., 2020) problems, though their use is not widespread. There are many methods for solving MAB problems, though the most common family of approaches is based on upper confidence bounds (UCBs). UCBs have been applied to Gaussian processes to map a scalar field over an environment (Luo and Sycara, 2018; Shi et al., 2020) and as the basis for a distributed Monte Carlo tree search algorithm for active perception (Best et al., 2019). Another MAB solution is Thompson sampling [TS; (Thompson, 1933)], which has recently proven successful in solving MAB problem in a stochastic manner (Russo et al., 2017). In fact, Chapelle and Li (2011) show that TS is among the most effective and easy-implemented MAB solvers algorithms. TS also allows for delayed feedback after sampling, which best fits distributed MRS scenarios since robots do not receive rewards until they reach their goal. In this paper, we choose to use a dynamic variation of TS (Gupta et al., 2011) for active target search, which handles the temporal variations of the target distribution.

### 1.3 Contributions

In this work, we develop a novel control policy that enables robots to actively gathering information, i.e., search for and track unknown targets, over a given task space. We have three primary contributions: 1) we introduce a distributed active search algorithm based on dynamic TS, 2) we combine the TS-based search with Lloyd’s algorithm for active tracking, 3) we propose a goal swapping algorithm to more effectively assign goals to each robot, and 4) we demonstrate in a series of simulated experiments that a team of robots using the combined TS and Lloyd’s algorithms more effectively finds and tracks targets than a team that uses only Lloyd’s algorithm.

The remainder of this paper is structured as follows. In Section 2, we address the problem of distributed target search and tracking, introduce the proposed distributed control strategy for idle robots to actively explore the environment, and present a novel algorithm that allows coordination of idle robots and tracking robots in looking for and tracking targets. In Section 3, we validate the proposed algorithms through a series of simulation results. Finally, we conclude in Section 4.

## 2 Method

### 2.1 Problem formulation

A set of nt targets with states $X=\left\{x_{1},\ldots ,x_{nt}\right\}$ are located within a convex open task space denoted by *E* $\subset R^{2}$. A team of *nr* (possibly heterogeneous) robots $R=\left\{r_{1},\ldots ,r_{nr}\right\}$ are tasked with determining *nt* and *X*, both of which are unknown and may vary over time. We assume that each robot knows its location $q_{i}$ in a global reference frame (*e.g.*, from GPS), though our proposed method can be immediately extended to handle localization uncertainty using the algorithms from our previous work (Chen and Dames, 2023). At each time step, a robot receives a set of noisy measurements $Z_{i}$ of targets within the field of view (FoV) of its onboard sensor. Note that the sensor may experience false negative or false positive detections so the number of detections may not match the true number of targets.

#### 2.1.1 PHD filter

The sets X and $Z_{i}$ from above contain a random number of random elements, and thus are realization of random finite sets [RFSs; (Mahler, 2007)]. The first order moment of an RFS is known as the *Probability Hypothesis Density* (PHD) (which we denote v (x)) and takes the form of a density function over the state space of a single target or measurement. The PHD filter recursively updates this target density function in order to track the distribution over target sets (Mahler, 2003).

The PHD filter uses three models to describe the motion of the targets: 1) The motion model, f(x|ξ), describes the likelihood of an individual target transitioning from an initial state ξ to a new state x. 2) The survival probability model, $p_{s}\left(x\right)$, describes the likelihood that a target with state x will continue to exist from one time step to the next. 3) The birth PHD, b(x), encodes both the number and locations of the new targets that may appear in the environment.

The PHD filter also uses three models to describe the ability of robots to detect targets: 1) The detection model, $p_{d}(x|q)$, gives the probability of a robot with state q successfully detecting a target with state x. Note that the probability of detection is identically zero for all x outside the sensor FoV. 2) The measurement model, g(z|x, q), gives the likelihood of a robot with state q receiving a measurement z from a target with state x. 3) The false positive (or clutter) PHD, c(z|q), describes both the number and locations of the clutter measurements.

Using these target and sensor models, the PHD filter prediction and update equations are shown as Equations 1–4:

(1) $${\bar{v}}_{t}\left(x\right)=b\left(x\right)+\int_{E}f(x|\xi )p_{s}\left(\xi \right)v_{t-1}\left(\xi \right)\;d\xi$$

(2) $$v_{t}\left(x\right)=(1-p_{d}(x|q)){\bar{v}}_{t}\left(x\right)+\sum_{z\in Z_{t}}\frac{\psi_{z,q}\left(x\right){\bar{v}}_{t}\left(x\right)}{\eta_{z}\left({\bar{v}}^{t}\right)}$$

(3) $$\eta_{z}\left(v\right)=c(z|q)+\int_{E}\psi_{z,q}\left(x\right)v\left(x\right)\;dx$$

(4) $$\psi_{z,q}\left(x\right)=g(z|x,q)p_{d}(x|q),$$

where $\psi_{z,q}\left(x\right)$ is the probability of a sensor at q receiving measurement z from a target with state x. The PHD filter recursively propagates the spatial density $v_{t}\left(x\right)$ of targets over time through prediction and update steps, accounting for target motion, births, deaths, and sensor measurements. In our implementation, we use the distributed version from (Dames, 2020) where each robot maintains the PHD within its Voronoi cell $V_{i}$. Three algorithms then account for motion of the robots (to update the subsets $V_{i}$), motion of the targets (in (1)), and measurement updates (in (2)).

#### 2.1.2 Lloyd’s algorithm

Lloyd’s algorithm minimizes the value of the functional shown as Equation 5:

(5) $$ℋ\left(\left\{q_{1},\ldots ,q_{nr}\right\}\right)=\int_{E}\underset{r\in R}{\min}f\left(d\left(x,q_{r}\right)\right)\phi \left(x\right)\;dx,$$

where d(x,q) measures the distances between elements in *E*, f(·) is a monotonically increasing function, and ϕ(x) is a non-negative weighting function. We use $f\left(x\right)=x^{2}$, a standard choice. The minimum inside of the integral induces a partition on the environment $V_{r}=\{x|d\left(x,q_{r}\right)\leq d\left(x,q_{i}\right),\forall i\neq r\}$. This is the Voronoi partition, and these $V_{r}$ are the Voronoi cells. Cortes et al. (2004) show that the gradient of (5) with respect to the state of each robot is independent of the states of the other robots, and that iteratively moving each robot r to its weighted centroid, shown as Equation 6:

(6) $$q_{r}^{*}=\frac{\int_{V_{r}}x\phi \left(x\right)dx}{\int_{V_{r}}\phi \left(x\right)dx},$$

achieves a local minimum of ℋ in a distributed manner. The control input for robot r is then $u_{r}\left(q_{r}^{*}\right)$, shown as Equation 7 where

(7) $$u_{r}\left(g\right)=\text{min }(d_{\text{step}},\|g-q_{r}\|)\frac{g-q_{r}}{\left\|g-q_{r}\right\|},$$

g is an arbitrary goal location, and $d_{\text{step}}>0$ is the distance a robot can move during one time step. By following this control law, robots asymptotically converge to the weighted centroids of their Voronoi cells. Note that Lloyd’s algorithm assumes a convex environment, though this restriction has been lifted in recent works (Breitenmoser et al., 2010) to allow for exploration in environments with obstacles. Our previous work effectively coupled tracking and control by using the distributed PHD as the weighting function (*i.e.*, ϕ(x)=v(x)) and using the Voronoi cells as the subsets $V_{i}$ (Dames, 2020).

### 2.2 Distributed Bernoulli Thompson sampling

While our prior work allowed robots to effectively track any detected targets, individuals do not actively search for a target and could often spend a long time locating targets that appeared in underexplored regions of the environment. This paper addresses this flaw in our prior work by proposing a new strategy for target search based on Thompson sampling. To do this, we divide the team into two subteams, a searching team $R_{s}=\{r\in R|Z_{r}=\emptyset \}$ (idle robots) and a tracking team $R_{t}=R\setminus R_{s}$ (busy robots), based on whether a robot is actively tracking at least one target. Tracking robots use the same Lloyd’s algorithm-based controller from (Dames, 2020) while searching robots use an active search strategy based on a novel distributed Bernoulli Thompson sampling method, detailed below.

#### 2.2.1 Modeling search as a MAB problem

We formulate the search task as a multi-armed bandit (MAB) problem, where the players (*i.e.*, robots) select actions among *K* resources with the goal of maximizing the expected reward. The number of resources, *K*, is fixed and finite, and a player instantly receives a reward $rw_{k}$ after completing an action k∈{1,…,K}.

In our context, each action k represents traveling to a specific region of the environment and the reward policy is a binary value (*i.e.*, we have Bernoulli bandits) based on the observation received at that location, shown as Equation 8:

(8) $$rw_{k}=\{\begin{array}{ll} 0 & Z=\emptyset \\ 1 & \text{else} \end{array},$$

**Figure 1:**
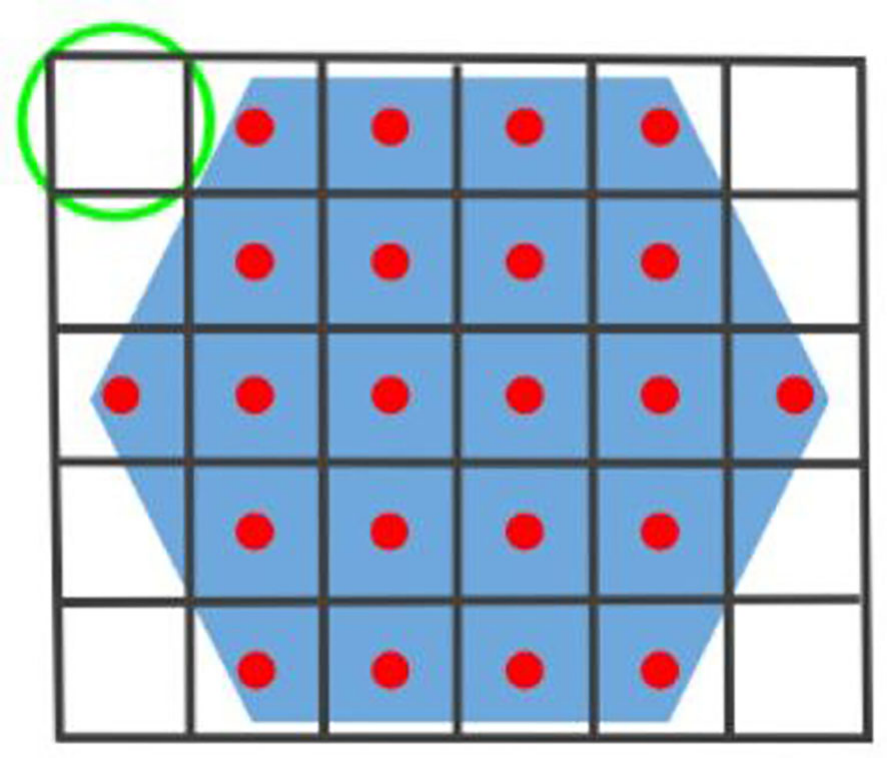
The hexagonal blue environment *E* is segmented by a set of black squares, each of which can be inscribed in a sensor FoV (green circle). The set of actions is represented by the red dots.

*i.e.*, the reward is 0 if the robot detects nothing at the goal location and 1 otherwise. To partition the environment *E* into *K* fixed regions, we assume that sensors are homogeneous and isotropic with FoV radii $\rho_{f}$. We then find the edge length of the inscribed square, *i.e.*, $\sqrt{2}\rho_{f}$. We tile these squares to completely cover *E*, discarding any squares that lie outside of the environment, as **Figure 1** shows. The center of each resulting square corresponds to a sampling position $s_{k}$ and the set of actions is then $S=\left\{s_{1},\ldots ,s_{K}\right\}$. This segmentation is conducted prior to the search task and is known by all robots.

There are several important points to note. First, our formulation is slightly different from the traditional MAB problem in that rewards are not immediately received after selecting an action. Instead, there is a time delay between selecting an action and receiving a reward due to the time it takes for a robot to travel to its goal $s_{k}$. Second, any inscribed polygon that can create a regular planar tiling would also work (*i.e.*, equilateral triangle or regular hexagon). Third, our approach can be extended to handle anisotropic or heterogeneous sensors by applying the similar strategy from our recent work (Chen and Dames, 2021), where we map each sensor to an isotropic FoV with equivalent detection capability then setting $\rho_{f}={\text{min}}_{r\in R}\rho_{f,r}$.

#### 2.2.2 Thompson sampling

Thompson sampling (TS), also known as posterior sampling, has proven successful in solving the MAB problem in recent decades (Russo et al., 2017). A Bernoulli bandit generates either a zero or a positive unit reward, *i.e.*, rw∈{0,1}, from each resource with a fixed and unknown probability $\theta_{k}\in \left[0,1\right]$. TS sequentially recommends an action for the next resource to sample from at each discrete time step t∈T using **Algorithm 1**, where beta(α,β) denotes the beta distribution with parameters α and β.

**Algorithm 1:**
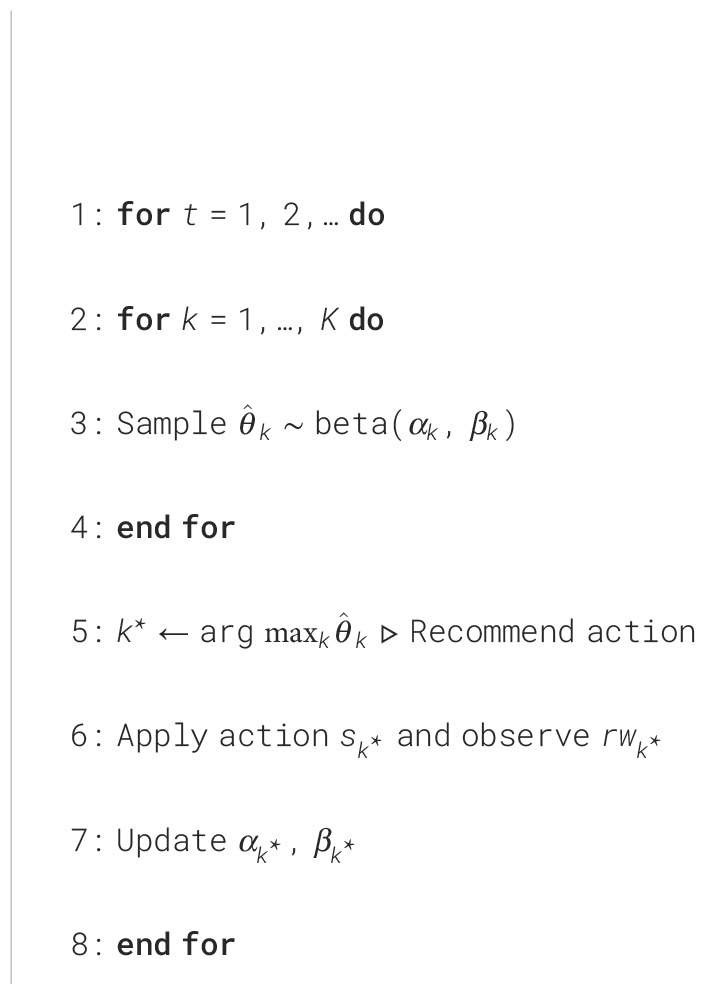
BernTS(*K*, *α*, *β*).

The algorithm takes the inputs of the number of resources K and initial parameters for the beta distributions for each resource, *i.e.*, $\alpha =\left[\alpha_{1},\ldots ,\alpha_{K}\right]$ and $\beta =\left[\beta_{1},\ldots ,\beta_{K}\right]$. At each discrete time step *t*, the reward posterior of each resource is sampled and the resource with the highest reward is selected (lines 2-5). The player takes the recommended action, receives the corresponding reward, and uses the reward to update the distribution parameters for the selected action (lines 6-7). The standard update equations for the parameters of the beta distribution are shown as Equation 9:

(9) $$\begin{array}{l} \alpha_{k}=\alpha_{k}+rw_{k}\\ \beta_{k}=\beta_{k}+\left(1-rw_{k}\right), \end{array}$$

where $\alpha_{k}$ and $\beta_{k}$ are called pseudo-counts since they increase by 1 after receiving a reward of 1 or 0, respectively (Russo et al., 2017). The beta functions then encode the posterior of getting a reward from each resource, allowing each resource to be sampled with a time-varying probability depending on current knowledge of the posterior distributions and resulting in an exploration behavior. As a growing number of observations are accumulated, the posterior distributions tend to congregate at true expected rewards and the recommended actions approach optimal, bringing about an exploitation behavior.

Traditional TS relies on a key assumption that the reward probabilities $\theta_{k}$ are fixed. However, this is not valid in our situation, where rewards depend on the presence on moving targets in certain regions of the environment. Using (9) can cause an irreversible loss of ability to react to future changes of reward probability. A variation of TS for dynamic reward probabilities was proposed by Gupta et al (Gupta et al., 2011), who replaced (9) with Equation 10:

(10) $$\begin{array}{l} \left(\alpha_{k},\beta_{k}\right)=\{\begin{array}{ll} \left(\alpha_{k}+rw_{k},\beta_{k}+\left(1-rw_{k}\right)\right), & \alpha_{k}+\beta_{k}<C\\ \frac{C}{C+1}\left(\alpha_{k}+rw_{k},\beta_{k}+\left(1-rw_{k}\right)\right), & \text{otherwise} \end{array}, \end{array}$$

where C is a constant threshold which $\alpha_{x_{t}}+\beta_{x_{t}}$ never exceeds. Such a strategy provides exponential weights to each update, weakening the impact from old samples so as to allow recent samples to dominate action selection. In this paper, we use (10) for beta distribution update in order to have robots to work in a potentially changeable environment.

Thompson sampling is also designed for a single player, however in our setting we have multiple cooperative players. To create a distributed TS algorithm, we maintain a consistent global α and β across the team, which each robot can use to independently sample actions when it enters the idle state. This requires robots to share their received rewards with one another, which happens once a robot reaches its goal location $s_{k^{*}}$. More specifically, after receiving a reward a robot will broadcast the tuple $\left(i,t,k^{*},rw\right)$ to its neighbors, which contains the robot ID, time stamp, action, and reward. Each robot then uses these tuples to update their local copies of α and β and rebroadcasts the tuple until all robots in the team have received it. This is guaranteed to happen in at most nr rounds since we assume that the communication graph is connected.

### 2.3 Multi-target information gathering

Our final control algorithm combines Lloyd’s algorithm and distributed Thompson sampling. Robots toggle between these two behaviors depending on whether they are busy or idle, *i.e.*, whether they are actively tracking a target, which we measure using Z≠∅. In the busy state, a robot uses the standard Lloyd’s algorithm, which effectively follows any targets within the current field of view. In the idle state, a robot uses TS to select a region to explore in search of a new target. Our formulation of the TS algorithm will bias robots to search areas which were recently known to contain targets while still allowing robots to visit unexplored regions. To enable a distributed coordination framework, we denote Remark 1.

Remark 1 (Distributed Voronoi Construction). *By assuming that each sensor is aware of the boundaries of* E*, and that the robot team contains more than one robot node and is connected*, $V_{r}$*can be constructed iteratively in a distributed fashion using***Algorithm 2***in* (Bash and Desnoyers, 2007) *that computes provably exact Voronoi cells without necessarily contacting the entire members of the team. This method requires that the communication graph is sufficiently dense, typically requiring a communication range at least twice the sensing or environment size scale, to ensure neighbors can exchange necessary boundary information.*

**Algorithm 2** outlines our strategy, where $g_{i}$ is the goal for robot $r_{i}$ and $N_{i}$ are the neighbors of robot $r_{i}$ in the communication graph, *i.e.*, the set of robots that it can directly communicate with. The “ParFor” block indicates that all robots execute their internal logic concurrently and asynchronously. All robots are initialized with their goal as the current location and with $\alpha_{k}=\beta_{k}=1,\forall k\in \left\{1,\ldots ,K\right\}$ (lines 1-6). As robots explore, they receive measurements. At each time step, robots must exchange states with their neighbors in order to compute Voronoi cells $V_{i}$ and update the distributed PHD (lines 9-12). Robot also use the measurements to update the beta distribution parameters for the nearest action point and broadcasts this information to all neighbors in the communication graph to ensure that all robots have identical information (lines 13-16). Once a robot reaches its goal, each robot will then enter either the idle or busy state, depending on the reward received, and select its next goal. In the idle state a robot uses the TS algorithm from Sec. 2.2 (lines 19-20), while in the busy state a robot uses Lloyd’s algorithm (6) (lines 22-23). If a robot is in the idle state, it has the option to swap goal with a neighbor in order to decrease the total distance traveled by the team (lines 26-28), the details of which are described in the next subsection. Finally, each robot moves towards it current goal (lines 29-30).

**Algorithm 2:**
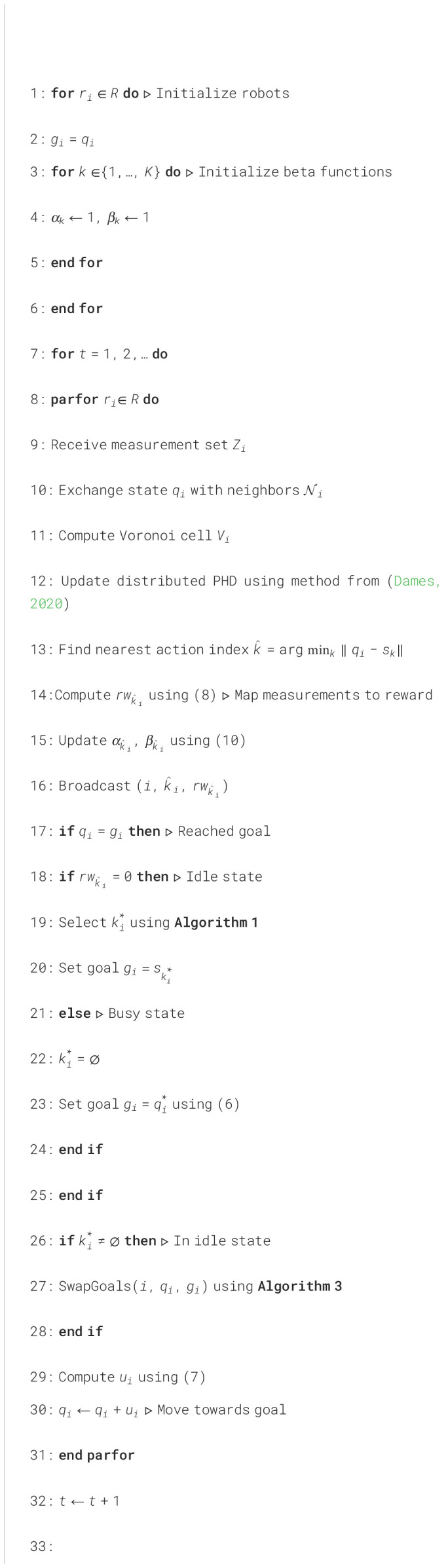
Distributed Search and Tracking.

#### 2.3.1 Optimized goal assignment

Since actions sampled from the TS algorithm can be anywhere in the environment, it is possible that robots may select points in inefficient ways, *e.g.*, two robots swapping places. To remedy this, we propose a goal reassignment strategy in which robots swap goals with a neighbor if both robots are in the idle state and if the swap will decrease the total distance traveled by the team, *i.e.*, $\exists j\neq i\text{ s}.\text{t}.\;d\left(q_{i}-g_{j}\right)<d\left(q_{j}-g_{i}\right)$. Once a goal swap occurs, the swapped robot continues to check the necessity of goal swapping in its neighborhood, as outlined in **Algorithm 3**. As a result of this, the distance between a robot and an assigned goal is always closer than the other pairs.

**Algorithm 3:**
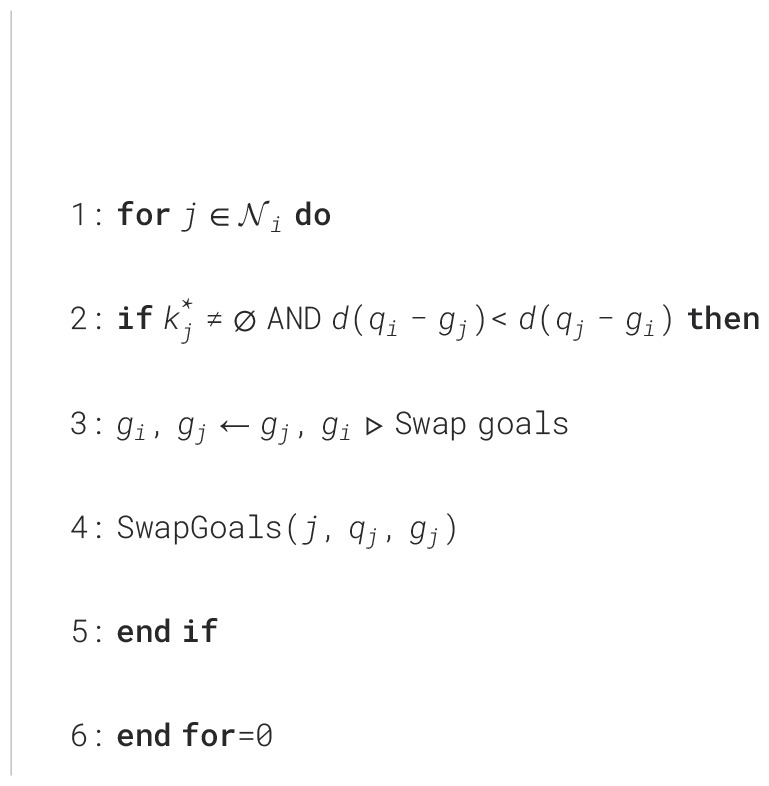
SwapGoals(*i*, $q_{i}$ , $g_{i}$ ).

Remark 2 (Communication Load). *In a Voronoi diagram, the average number of neighbors for a cell is bounded by 6, though it can vary* (Boots et al., 1999)*. Therefore, at each time step, a robot* $r_{i}$*exchanges* $T=\left\{q_{i},i,{\hat{k}}_{i},rw_{{\hat{k}}_{i}},g_{i}\right\}$*and partial PHD (***Algorithm 2***, line 12), i.e., PHD within its partition, with limited number of neighbors. Since* T*is an array with length of 6 in a 2D* sp*ace and the partial PHD is of low bandwidth referred to* (Dames, 2020)*, our algorithms requires only low communication load within neighborhoods.*

## 3 Results

**Algorithm 3** boosts the performance of our previous methods (Dames, 2020) in that it allows the team to actively explore the environment and learn the characteristics of the target distribution. In particular, robots are now able to use a combination of detailed local information (coming from the PHD) and coarse global information (coming from α,β) to inform their actions. The advantages of adding this global information are especially pronounced when targets are not uniformly distributed in the space but are instead grouped together within small regions. Under these circumstances, idle robots are especially helpful in learning the difference in target density among sub-regions and optimizing the assignment of tracking effort in different sub-regions.

### 3.1 Simulation environment

We test our proposed algorithms via Matlab simulations. The task space is a 100 × 100 m square. Targets may either be static or moving within their sub-regions at a maximum speed of 3 m/s. Existing targets may disappear (*i.e.*, leave the environment) and new targets may appear (*i.e.*, enter the environment) so that the number of targets may change over time.

All robots begin each trial at randomized locations within a 20 ×10 m box at the bottom center of the environment. Robots have a maximum speed of 10 m/s and are equipped with isotropic sensors with a sensing radius $\rho_{f}=5$ m. Thus, the environment is segmented into a grid of 14 ×14 points, using the method from Sec. 2.2.1. We use C=10 in (10).

In the PHD filter, we assume the robots do not have any prior knowledge of the targets. Thus, the robots use a Gaussian random walk (with σ=0.35 m/s) for the motion models *f*, set the survival probability to 1, and the birth PHD to 0. We use the same measurement model for sensors as in (Dames, 2020), with the exception of assuming that sensors are all homogeneous and produce no missed or false detections. Note that our proposed method is compatible with heterogeneous sensing network (Chen et al., 2025a) and false alarms (Dames, 2020), we just make this choice to simplify the tests and highlight the improvement relative to our previous method.

### 3.2 Qualitative comparison

**Figure 2:**
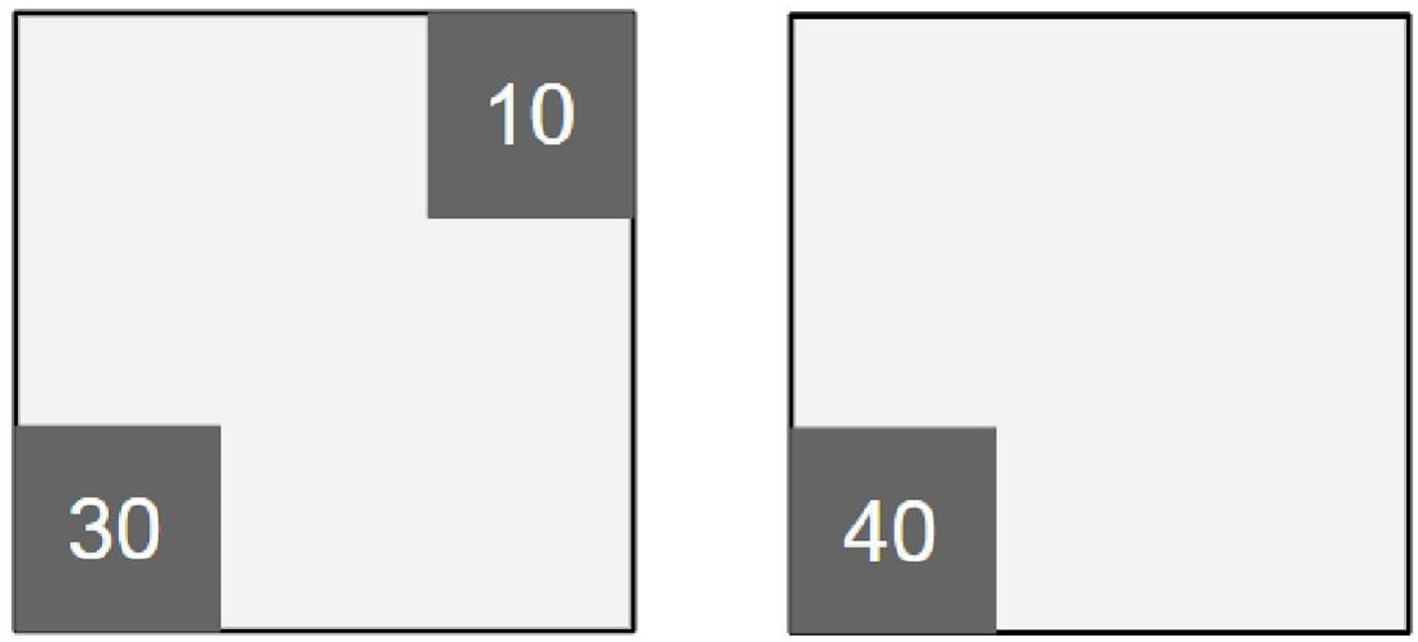
Figures show target distributions in two different environments. Black lines are boundaries of the 100 m × 100 m task spaces. Targets are only distributed within 33 m × 33 m dark gray areas and the numbers indicate the original numbers of targets in these areas respectively.

We first show how active search using TS qualitatively improves multi-target tracking using a single trial. There are 40 robots searching for 40 targets, where 30 targets are located in a 33 × 33 m square sub-region at the lower-left corner of *E*, and another 10 targets in a 33 × 33 m squared sub-region at the top-right corner, as shown in **Figure 2a**. Targets locations are drawn uniformly at random within each sub-region. For simplicity, the targets are stationary and the number of targets is constant over time (note: the robots still use a Gaussian motion model within the PHD filter).

**Figure 3:**
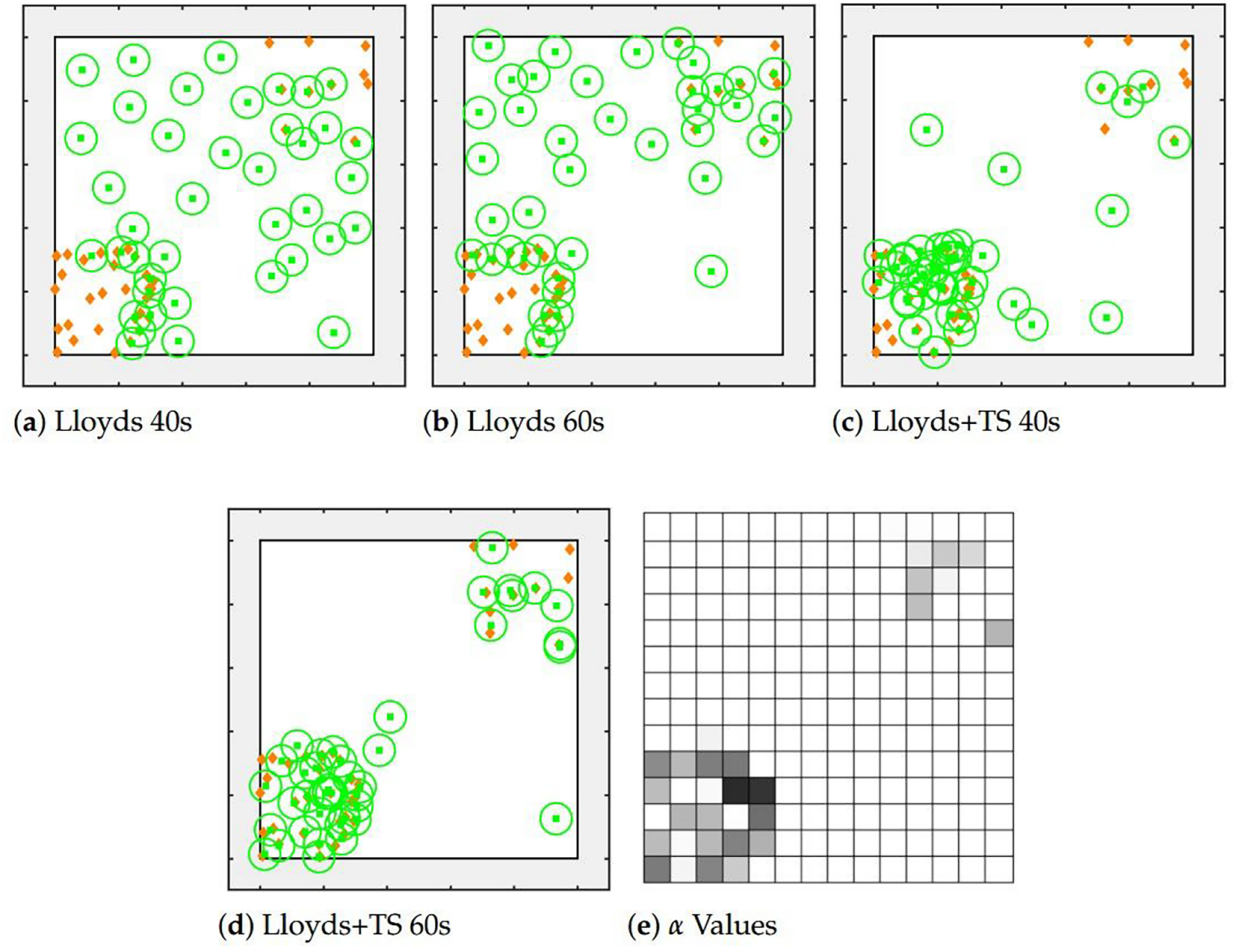
Figures show comparison of applying pure Lloyd’s algorithm and a combined Lloyd’s algorithm with Thompson sampling after 100 s. In **(a-d)**, green squares and circles show robot locations and sensor footprints, respectively. Orange diamonds show the locations of targets. **(e)** maps *α* values in **(d)** by darkness, with a darker color indicating a higher value.

**Figure 3** shows the locations of robots and targets at various points during exploration using both our previous (Dames, 2020) and new methods. When using our previous method, which only used Lloyd’s algorithm, a large portion of robots are idle even when a fair amount of targets are not tracked after 40 s, as the centroids of these orange diamonds are not located in any of the green circles. After 60 s, robots tend to move towards the two corners with targets but a large portion of them have still not found any targets. We also see that robots get stuck on the boundary of target clusters, never reaching the interior targets. The result demonstrates the weakness of pure Lloyd’s algorithm that idle robots do not actively search for targets, causing an inefficient use of the total sensing capability of the team while searching for untracked targets.

On the other hand, the team using distributed Thompson sampling is able to quickly learn the target distribution and cluster in regions likely to contain targets. As the figure shows, after 40 s a large number of the robots have already gathered at the large cluster of targets while a handful of other robots continue to search unexplored areas. After 40 s, most of the robots have found a target while a few continue to maintain coverage of these unlikely regions. The resulting emergent robot distribution is consistent with the target distribution, as the lower-left corner contains a higher number of robots than the upper-right corner (28 vs. 9, which closely match the number of targets in each region), while 3 robots monitor the rest of the area. This helps the team to track the targets more quickly as the more individuals are needed in an area, the more likely it is that an individual will be assigned to that region. The final panel reflects the value of α for each sampling candidates after 60 s, showing that the team has received more reward in areas with higher target concentrations.

### 3.3 Quantitative comparison

To quantify the improvement in performance, we will use the first order Optimal SubPattern Assignment (OSPA) metric (Schuhmacher et al., 2008), a commonly-used approach in MTT. The error between two sets *X, Y*, where |X|=m≤|Y|=n without loss of generality, is Equation 11

(11) $$d\left(X,Y\right)={\left(\frac{1}{n}\underset{\pi \in {\text{Π}}_{n}}{\min}\left(\sum_{i=1}^{m}d_{c}{\left(x_{i},y_{\pi \left(i\right)}\right)}^{p}+c^{p}\left(n-m\right)\right)\right)}^{1/p},$$

where c is a cutoff distance, $d_{c}\left(x,y\right)=\text{min }(c,\|x-y\|)$, and ${\text{Π}}_{n}$ is the set of all permutations of the set {1,2,…,n}. This gives the average error in matched targets, where OSPA considers all possible assignments between elements x∈X and y∈Y that are within distance c of each other. This can be efficiently computed in polynomial time using the Hungarian algorithm (Kuhn, 1955). We use c=10 m, p=1, and measure the error between the true and estimated target sets. Note that a lower OSPA value indicates a more accurate tracking of the target set. We report the median OSPA value over the final 150 s of each trial, allowing the team to reach a steady state and smoothing out the effects of spurious measurements that cause the OSPA to fluctuate. We also show the 95% rise time of the OSPA error metric, *i.e.*, the time it takes for the OSPA error to reach a value within 5% of the final value, to measure the speed at which robots reach steady state.

#### 3.3.1 Tests design

To test the efficacy of our proposed approach, we conduct a series of trials in two environments, shown in **Figures 2a, b**, and in situations where targets are either all stationary or all dynamic (note: the robots always use the same target models in the PHD). For each environment and each target motion type, we test a range of team sizes (from 10 to 40 robots) and both search strategies (from (Dames, 2020) and the new method). For each configuration (environment, target type, team size) we run 10 trials, with the results aggregated into boxplots showing the steady state OSPA (to measure accuracy) and the 95% rise time (to measure speed).

**Figure 4:**
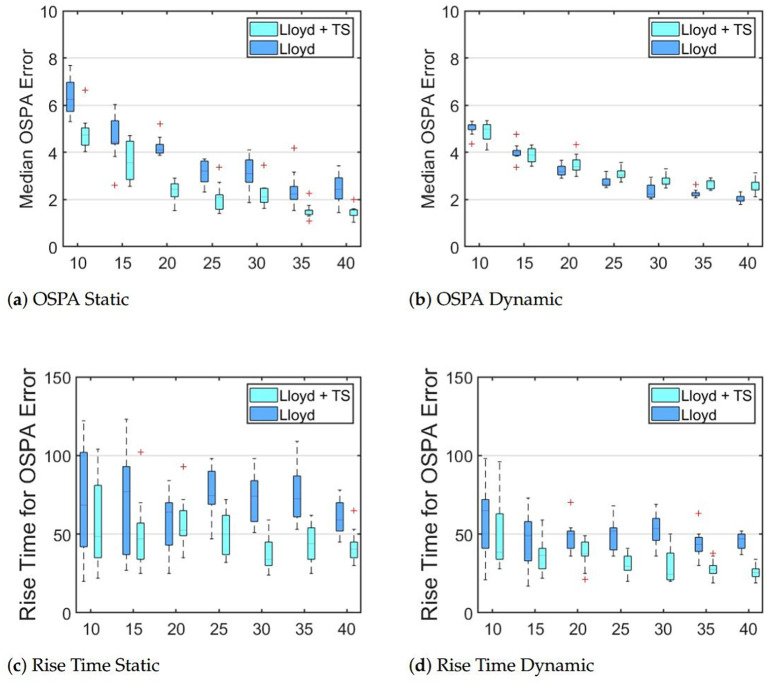
Boxplots show median OSPA errors and the 95% rise time for robot teams tracking targets distributed as in **Figure 2a**.

**Figure 4** shows the results from the first environment (**Figure 2a**). As we see in the OSPA plots, the median OSPA decreases as the number of robots increases. This agrees with intuition as more robots should be able to better locate targets and there should be diminishing rewards with each added robot, *i.e.*, going from 10 to 15 robots is more significant than 35 to 40 robots. We see that for static targets, our proposed method shows significantly lower OSPA error and rise time and that the variation of both parameters across trials is smaller, meaning it more accurate, faster, and more reliable. For dynamic targets, the OSPA error of teams using our proposed method are comparable or slightly higher and exhibit slightly more variation across trials, though neither effect is significant. Like the static case, our new method decreases both the magnitude and variation of the rise time, meaning it faster and more repeatable. We hypothesize that these differences in behavior are due to the ability of robots to actively sample the environment using coarse global information. We also believe that the primary cause of the slight increase in OSPA error in the case of dynamic targets is because robots do not exit the idle state until they reach their destination. This means that if a robot observes a target en route to its goal, it will continue towards the goal instead of actively tracking the newly found target.

**Figure 5:**
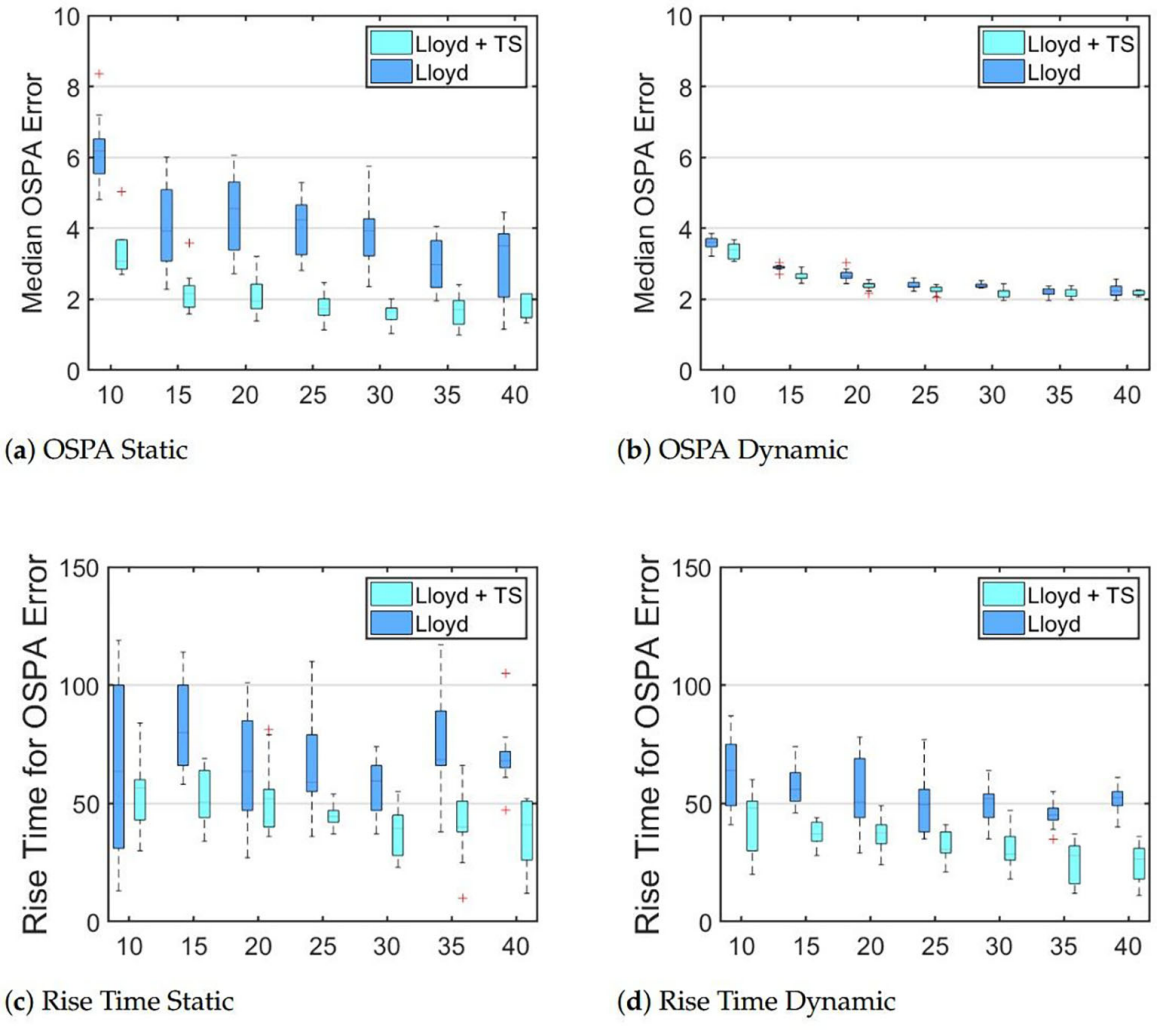
Boxplots show median OSPA errors and the 95% rise time for robot teams tracking targets distributed as in **Figure 2b**.

**Figure 5** shows the results in the second environment (**Figure 2b**). The results are consistent with those from previous tests, except we now see a slight improvement in OSPA error for dynamic targets. We believe this is due to the high density of targets reducing the total cost of moving towards sampling points. We also see that the reduction of the rise time is more pronounced in every case than it was in **Figure 4**, supporting our argument that the proposed control algorithm is more beneficial when targets are more tightly congregated.

## 4 Conclusions

We develop an active information gathering algorithm for distributed MRSs combining a novel distributed Thompson sampling algorithm with Lloyd’s algorithm to allow robots to effectively search for and track multiple moving targets without having any prior knowledge about targets. In particular, we see that the addition of TS allows robots to share coarse global information about recently detected targets in an efficient and scalable manner. As a result, teams using TS are able to more accurately track targets, locate targets more quickly, and increase the consistency in performance. These trends are more pronounced in situations where targets are unevenly distributed within the search space.

## Data Availability

The raw data supporting the conclusions of this article will be made available by the authors, without undue reservation.
