# Correction: Distributed multi-robot active gathering for non-uniform agriculture and forestry information

**DOI:** 10.3389/fpls.2025.1730134

**Published:** 2025-11-06

**Authors:** Jun Chen, Mingjia Chen, Jun Wang, Qi Mao, Fei Xie, Philip Dames

**Affiliations:** 1 Wenzhou Vocational College of Science and Technology, Wenzhou, China; 2 Wenzhou Key Laboratory of AI Agents for Agriculture, Wenzhou, China; 3 School of Electrical and Automation Engineering, Nanjing Normal University, Nanjing, China; 4 Department of Mechanical Engineering, Temple University, Philadelphia, PA, United States

**Keywords:** multi-robot systems, active information gathering, Thompson sampling, multi-target tracking, distributed control

The title of this article was erroneously given as “Distributed multi-robot active gathering for non-uniform agriculture and forestry information multi-robot active gathering”. The correct title of the article is “Distributed multi-robot active gathering for non-uniform agriculture and forestry information”.

The original version of this article has been updated.

